# Rate of Post-Operative Pancreatic Fistula after Robotic-Assisted Pancreaticoduodenectomy with Pancreato-Jejunostomy versus Pancreato-Gastrostomy: A Retrospective Case Matched Comparative Study

**DOI:** 10.3390/jcm10102181

**Published:** 2021-05-18

**Authors:** Marco V. Marino, Adrian Kah Heng Chiow, Antonello Mirabella, Gianpaolo Vaccarella, Andrzej L. Komorowski

**Affiliations:** 1 Department of General and Emergency Surgery, Azienda Ospedaliera Ospedali Riuniti Villa Sofia-Cervello, 90127 Palermo, Italy; antonellomirabella@gmail.com (A.M.); janpaol@yahoo.it (G.V.); 2 Department of General and Oncologic Surgery, Università degli Studi di Palermo, 90127 Palermo, Italy; 3 Hepatopancreatobiliary Unit, Department of Surgery, Changi General Hospital, Singapore 529889, Singapore; adrian.chiow.k.h@singhealth.com.sg; 4 Department of General Surgery, College of Medicine, University of Rzeszow, 35-959 Rzeszow, Poland; z5komoro@cyf-kr.edu.pl

**Keywords:** robotic pancreatic surgery, pancreato-gastrostomy, pancreatic fistula

## Abstract

Background: Different techniques of pancreatic anastomosis have been described, with inconclusive results in terms of pancreatic fistula reduction. Studies comparing robotic pancreaticogastrostomy (PG) and pancreaticojejunostomy (PJ) are scarcely reported. Methods: The present study analyzes the outcomes of two case-matched groups of patients who underwent PG (*n* = 20) or PJ (*n* = 40) after pancreaticoduodenectomy. The primary aim was to compare the rate of post-operative pancreatic fistula. Results: Operative time (375 vs. 315 min, *p* = 0.34), estimated blood loss (270 vs. 295 mL, *p* = 0.44), and rate of clinically relevant post-operative pancreatic fistula (12.5% vs. 10%, *p* = 0.82) were similar between the two groups. PJ was associated with a higher rate of intra-abdominal collections (7.5% vs. 0%, *p* = 0.002), but lower post-pancreatectomy hemorrhage (2.5% vs. 10%, *p* = 0.003). PG was associated with a lower rate of post-operative pancreatic fistula (POPF) (33.3% vs. 50%, *p* = 0.003) in the high-risk group of patients. Conclusions: The outcomes of post-operative pancreatic fistula are comparable between the two reconstruction techniques. PG may have a lower incidence of POPF in patients with high-risk of pancreatic fistula.

## 1. Introduction

Pancreaticoduodenectomy (PD) is a complex operation associated with significant post-operative mortality (1–6%) [1] and morbidity rate (10–45%) [2], even at high-volume pancreatic centers [3].

The management of the pancreatic remnant is still controversial, and multiple reconstructive techniques have been reported [4,5]. The main goal of each technique is to minimize the occurrence of post-operative pancreatic fistula (POPF), and its consequence on patient outcomes [6]. Pancreaticojejunostomy (PJ), including pancreatic invagination or duct-to-mucosa anastomosis [7,8], and pancreaticogastrostomy (PG) are the most commonly used reconstructive techniques [9]. Technical details are mainly based on surgeon’s preference, in the attempt to define the ideal technique to reduce POPF [5].

Current evidence does not show any conclusive advantage of one technique over another. To date, there are no specific recommendations on how to manage the pancreatic stump after pancreaticoduodenectomy [10]. Minimal invasive pancreatic surgery is gaining an increased interest worldwide both for distal pancreatectomy [11] and pancreaticoduodenectomy [12,13]. Robotic pancreaticoduodenectomy (RPD) may mitigate some risk factors of POPF, such as blood loss [14]. However, to date, few comparative studies have been performed comparing RPD with PG and PJ [15]. The present study aims to compare the post-operative outcomes of PG and PJ after RPD.

## 2. Materials and Methods

A retrospective analysis of a prospectively maintained database including all RPD carried out between August 2014 and October 2019 at the Department of General Surgery, of our Tertiary Care Center was performed.

Patients with a preoperative diagnosis of benign tumor or localized and resectable malignant tumor at the periampullary region who did not meet any of the exclusion criteria (Table 1) were selected for RPD and they were included in the study. All pancreatic anastomoses in RPD until 2018 were PJ. Subsequently, the PG anastomosis technique was adopted as the only method for pancreatic reconstruction during RPD. The same surgeon performed all the anastomoses during the time period of the study.

The study was approved by the Institutional Review Board of the hospital. Informed written consent was obtained from all participants and the study has been carried out following the declaration of Helsinki guidelines.

### 2.1. Study Endpoints

The primary outcome of the present study was to compare the effectiveness of the robotic PG reconstruction versus PJ in patients undergoing RPD in terms of POPF rate.

Secondary outcomes were the length of hospital stay, duration of surgical intervention, time needed to complete the pancreatic anastomosis, rate of surgical re-intervention and of overall post-operative complications. The study compared the results of patients who underwent PG (*n* = 20) and PJ (*n* = 40). The two groups were further case-matched using four variables, in accordance with the POPF scoring system of Callery et al. (soft pancreatic texture, disease pathology, pancreatic duct diameter <3 mm, intraoperative blood loss) [16,17,18]. Based on gland texture, pathology, pancreatic duct diameter, and intraoperative blood loss, the patients were scored according to the fistula risk score (FRS) from a total of 0–10 points. They were then subclassified into negligible risk (0 points), low risk (1–2 points), intermediate risk (3–6 points) and high risk (7–10 points) [16]. The patient population was also classified and case-matched according to the recent ISGPS classification for parenchyma risk factors proposed by Schuh et al. [19].

A risk analysis was performed to confirm all potential risk factors for POPF.

### 2.2. Definitions

POPF was defined and graded using the revised consensus guidelines by the International Study Group for Pancreatic Fistula (ISGPF) [20].

The pancreatic texture was assessed on the resected pancreatic specimens and classified as hard or soft. Pancreatic duct diameter was measured by intraoperative ultrasound and confirmed on the cutting surface of the remnant pancreas using a ruler.

Post-operative complications were graded according to the Clavien-Dindo classification system, and Grade III or higher were regarded as significant complications [21]. The highest grade of complication was considered in patients with more than one complication.

Biliary fistula, delayed gastric emptying, and post-pancreatectomy hemorrhage were classified using international definitions [22,23,24]. Intra-abdominal abscess or fluid collection were diagnosed based on post-operative ultrasound or computed tomography (CT) scans [25].

Operative time was defined as the time from skin incision to wound dressing. Intra-operative blood loss was quantified by measuring the amount of fluid obtained from the suction device.

Mortality was defined as a death that occurred within 90 days after surgery.

### 2.3. Surgical Technique

Our surgical technique for a fully robotic-assisted pylorus-preserving RPD was previously described elsewhere [26].

The PJ was fashioned with an end-to-side duct-to-mucosa two-layer anastomosis with interrupted sutures (Cattell Warren technique). A continuous 3/0 V-loc™ (Covidien; Mansfield, MA, USA) suture was placed between the seromuscular layer of the jejunum and the posterior capsule of the pancreatic remnant. Then, the jejunum was opened, and the pancreatic duct was secured to the jejunal mucosa using 5/0 polypropylene interrupted sutures (PROLENE^®^). A 3/0 V-loc™ self-fixating running suture finally approximated the anterior jejunal seromuscular layer and the anterior aspect of the pancreatic remnant (Supplementary Video S1 Part A).

For the trans-gastric PG anastomosis, a 2.5-cm longitudinal gastrostomy was performed on the anterior wall of the stomach, and the pancreas was invaginated into the gastric lumen through a small opening on the posterior gastric wall, enlarged to approximately half of the pancreatic diameter. The pancreatic remnant was pulled holding the stay sutures previously placed as described by Giulianotti et al. during robotic PD [27]. Then, the pancreatic parenchyma was sutured to the gastric mucosa using interrupted 4/0 polydioxanone (PDS II^®^) sutures. The anterior gastrotomy was closed with a 3/0 PDS running suture (Supplementary Video S1 Part B).

In all cases, an internal not secured 5-French (duct size < 4 mm) or 7-French (4–8 mm duct size) silastic pediatric feeding tube was inserted into the pancreatic duct to assure its patency.

Finally, an abdominal (12 French) closed-suction drain was placed behind the pancreatic anastomosis reaching also the anterior aspect of the hepaticojejunostomy.

### 2.4. Statistical Analysis

Continuous variables were expressed as mean values ± standard deviation (SD) or as median and interquartile range (IQRs) where appropriate. Categorical data were presented as frequency and percentages. Fisher’s exact test and Pearson Chi square test and the Mann-Whitney U test were used to define associations between categorical and continuous variables, respectively. Univariate analysis was carried out to identify all significant factors which have been reported to influence POPF: age, gender (male), body mass index >25 Kg/m^2^, diabetes mellitus, and cardiovascular disease [28,29].

SPSS 19.0 (SPSS Inc, Chicago, IL, USA) was used for the statistical analysis. A *p* value < 0.05 was considered statistically significant. Variables with *p* < 0.10 were included in the multivariate analysis.

## 3. Results

A total of 60 patients underwent RPD during the study period. Twenty patients underwent PG, while 40 patients underwent PJ. Table 2 shows the preoperative features and final pathology data. Pancreatic ductal adenocarcinoma was the most common indication for surgery (48.3%). In the same period, a total of 282 patients who underwent open PD did not meet the criteria for RPD.

Patients in the PJ and PG groups had similar risk factors for POPF development. The fistula risk score (FRS) was distributed as follows: eight patients (13.3%) had a negligible risk, 24 (40%) low risk, 21 (35%) moderate risk, and seven (11.7%) patients had high risk, without any difference between PJ and PG groups.

The overall operative time (median ± SD) was 355 min ± 103. Patients who underwent PG had similar operative time compared to PJ (315 vs. 375 min, *p* = 0.345).

The fashioning of PJ required a longer time in comparison to PG (32 ± 11 vs. 25 ± 14 min, *p* = 0.002). The median (IQR) estimated blood loss was 275 mL (180–600). No statistically significant difference was observed between the two groups (270 vs. 295 mL, *p* = 0.442).

A total of seven patients experienced a clinically significant POPF (11.7%), with a similar rate after PG and PJ (12.5% vs. 10%, *p* = 0.820).

A 18.3% rate of severe complications was reported, with the two group of patients showing a similar morbidity rate (20% vs. 15%, *p* = 0.542). Two patients in the PJ group underwent a reoperation due to the onset of clinically relevant POPF and ascites which required the disassembly of the pancreatic anastomosis and the fashioning of a new PJ. In the PG group, a patient with post-operative bleeding required a surgical revision after the failure of endoscopic approach.

The post-operative hospital stay was comparable between the two groups (14 ± 4 vs. 11 ± 6 days, *p* = 0.223). The overall mortality rate was 5% (Table 3).

The case-matched analysis according to the four variables of the clinical risk score for POPF (soft pancreatic texture, pancreatic duct diameter < 3 mm and intraoperative blood loss > 500 mL and histopathology), showed that PJ was associated with longer anastomotic time (46 vs. 25 min, *p* = 0.002), but not with an increased risk of POPF. PJ was associated with a higher rate of intrabdominal collection (*p* = 0.002), but a lower rate of post-pancreatectomy hemorrhage (*p* = 0.003) (Table 4).

**Figure 1:**
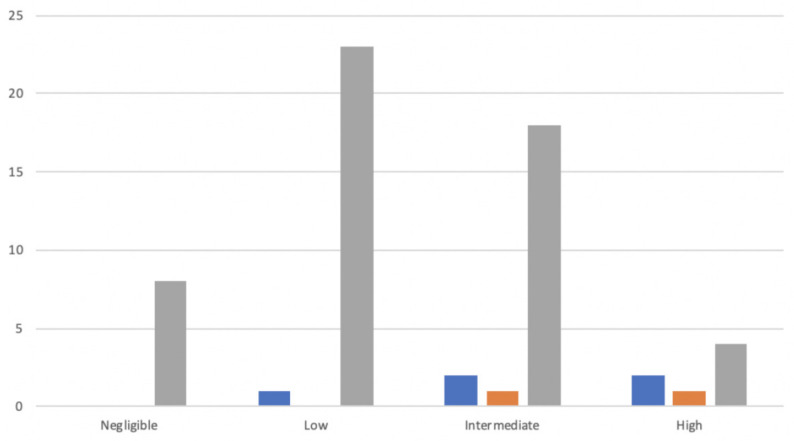
Rate of the clinically relevant postoperative pancreatic fistula (CR-POPF) in a subgroup analysis.

In the univariate analysis, risk factors for POPF were BMI, pancreatic duct diameter, the texture of the pancreas, and estimated blood loss. PJ was not associated with an increased risk of POPF (Table 5). Three out of seven patients experienced a CR-POPF in the high-risk group (Figure 1).

In the stratified analysis according to the clinical risk score, there was no significant difference for cases included in the low-risk group (PG 0% vs. PJ 6.3%, *p* = 0.445) and intermediate-risk (PJ 14.3% vs. PG 14.3%, *p* = 1.000) in terms of POPF. In contrast, PG was associated with a lower rate of POPF in the high-risk group (PG 33.3% vs. PJ 50%, *p* = < 0.05).

At six months follow-up, three patients in the PG group were readmitted for vague abdominal pain, dyspepsia, abdominal distension associated with changes in bowel habit. No sign of anastomotic stricture was observed during the diagnostic tests.

In the PJ group we observed two hospital readmissions in patients experiencing fever and associated fatigue. In both cases, an abdominal collection was detected at diagnostic CT-scan.

## 4. Discussion

Despite significant advancements in the operative techniques and improvements in perioperative surgical care, more than 20% of patients still develop a POPF after PD [30].

To date, there is no gold standard technique for pancreatic anastomosis and experience-related methods are connected to the surgeon expertise, so that “the best anastomosis is probably the one with which the surgeon is most familiar”.

A recent systematic review comparing open PG versus PJ concluded that the two techniques are equivalent in terms of overall post-operative outcomes, nevertheless PJ seemed associated with a slight reduction of post-operative bleeding (9.3% vs. 13.8%), but it showed a higher risk of developing intra-abdominal abscess (14.7% vs. 8.0%) compared to PG [10]. This was also a consistent finding in our study.

Open PD still represents the gold standard in case of resectable pancreatic head tumors, whereas minimally invasive PD is currently performed in selected high-volume centers [31].

In our experience, the decision for the shift in the reconstruction strategy from the conventional PJ technique to the PG has to be found in the emerging evidence from multicenter randomized controlled trials showing that the incidence of POPF is lower in patients undergoing PG than in those undergoing PJ [32,33]. However, our results showed that both techniques are equally feasible and safe, with similar morbidity rate and length of in-hospital stay.

Significant efforts have been directed at identifying risk factors of POPF after PD. Callery et al. validated a model for predicting clinically significant POPF after PD by using four parameters: pancreatic texture, pathology, pancreatic duct diameter, and intra-operative blood loss which are incorporated into a convenient scoring system of risk categories [16].

Some risk factors, such as soft pancreatic texture, small pancreatic duct diameter and higher BMI are no modifiable because they are inherent to the patient. Conversely, the anastomotic technique is the only factor that can hypothetically modify the risk of POPF after PD. In the present study, the rate of POPF after PG and PJ was not statistically different overall, although subgroup analysis showed lesser POPF in PG in patients with high FRS.

A broad Cochrane systematic review published in 2017 concluded that PG may have little or no difference compared to PJ in the overall risk of any surgical complications and particularly in POPF formation, mortality and length of post-operative hospital stay, concluding that there was no reliable evidence to support the use of PG over PJ [10].

A recent meta-analysis including 11 RCTs that enrolled a total of 1765 patients concluded that POPF was related to a significantly lower morbidity rate in the PG group than in the PJ group (OR = 0.67, *p* = 0.002). In contrast, clinically significant POPF rates were not significantly different between the two groups (OR = 0.61, *p* = 0.09). PJ was also associated with a statistically significant lower incidence of post-operative bleeding compared with PG (OR = 1.47, *p* = 0.03), whereas the rate of delayed gastric emptying was not significantly different (OR = 1.09, *p* = 0.54) [34].

The RPD is gaining momentum among the pancreatic surgeon community, and recently the correlation between robotic approach and POPF was investigated [35]. Although the benefits shown in terms of lower estimated blood loss and shorter length of hospital stay, RPD failed to demonstrate a significant reduction in the POPF compared to open PD [12,36].

A multi-institutional study using data from the American College of Surgeons National Surgical Quality Improvement Program concluded that patients undergoing minimally invasive PD had higher rates of clinically relevant POPF compared to open PD (15.3% vs. 13.0%, *p* = 0.03), but the surgical technique was not an independent factor associated with POPF on the adjusted multivariate analysis (OR 1.05, 95% CI 0.87–1–26) [37]. Conversely, in tha high volume center, the RPD was associated to lower CR-POPF when compared to OPD (6.7% vs. 15.8%, *p* < 0.001) [38].

The present study found similar results between the two anastomosis techniques in terms of operative time and estimated blood loss, although PJ required longer operative time for its fashioning. From a technical point view, the PJ was more challenging for the higher number of sutures required and for the difficulties in exposing the posterior row of the anastomosis. On the other hand, the PG required a major traction on the pancreatic stump that may cause bleeding from pancreatic surface. This may account for the higher rate of post-operative collection, but lower post-operative hemorrhage noted for PJ compared to PG in our study.

Since 2013, the fistula risk score was developed to assess the risk of clinically relevant POPF. While widely used, recent studies have found that not all factors were statistically significant especially with respect to blood loss suggesting that newer predictive models maybe necessary [18]. This includes alternative FRS comprising of pancreatic texture, pancreatic duct diameter and body mass index (BMI) [39]. Recently, Polanco et al. found that high BMI, high estimated blood loss, smaller tumor size and small duct diameter are the main predictors for POPF in RPD [40].

In the present study, the univariate analysis revealed that the pancreatic duct diameter, as well as soft consistency of the pancreas, higher BMI and higher blood loss, were associated with increased risk of POPF. The diameter of the pancreatic duct and the soft texture of the pancreas influenced the rate of POPF heavily, as demonstrated by the fact that 66.6% of patients who developed a POPF had a pancreatic duct diameter <3 mm and 55.6% of patients had a soft pancreatic texture. The soft pancreas is more susceptible to ischemia and injury. Moreover, soft texture is generally associated with a small pancreatic duct, and a preserved exocrine function, resulting in increased activation and secretion of pancreatic juice [41]. A narrowed pancreatic duct is not only more challenging to reconstruct, but anastomoses in such cases are also more likely to either occlude or dehisce [40]. In our study, the rate of POPF after PG is significantly lower in patients with high risk of POPF as the PG obviates the need to anastomose the pancreatic duct compared to the PJ duct to mucosa technique. Further studies are needed to determine better predictive models for POPF. A large adequately powered well designed RCT comparing PG versus PJ in robotic PD from experienced centers may be the next step to consider the validity of our findings and further shed light to the optimal method for reconstruction in this complex surgery.

Our retrospective cohort study has several limitations. It has been carried out at a single-institution and included a small cohort of patients. The case matched study design reduced the number of involved procedures in the analysis. Furthermore, the study compares only one type of PJ compared to PG and its findings may not be applicable to PJ reconstruction via other techniques. However, the bias related to variations in the surgical technique and the post-operative management is minimal as the same pancreatic team performed all the RPD in this series. A major experience and a growing number of cases performed by the team in the near future will add more validity to the conclusions drawn. A comparison among the outcomes of other experienced centers could lead to a standardization of this complex and emerging surgical technique.

## 5. Conclusions

The present study showed that the rate of POPF after robotic PG and PJ were equivalent with a lower rate of POPF after PG for patients at high risk of POPF.

## Figures and Tables

**Table 1:** Exclusion criteria from the study.

Unsuitability for pneumoperitoneum
ASA score > III
Body mass index (BMI) < 35 kg/m^2^
Borderline or Locally advanced tumours
Intraperitoneal or extraperitoneal metastases
Tumor size > 5 cm
Patients who underwent total pancreatectomy
Patients requiring concomitant organ or vascular resection
Conversion to open

Abbreviation: ASA, American Society of Anaesthesiologists.

**Table 2:** Demographic, pre-operative characteristics and risk factors variables for post-operative pancreatic fistula (POPF) of patient undergoing robotic pancreatojejunostomy (PJ) and pancreatogastrostomy (PG).

Variables	PJ (*n* = 40)	PG (*n* = 20)	Overall (*n* = 60)	*p* Value
Age, years, median (IQR)	63.2 (55.6–71.4)	61.9 (53.8–68.5)	62.9 (54.1–71.1)	0.688
Sex, *n* (%)				
Male	27 (67.5%)	13 (65%)	40 (66.7%)	0.627
Female	13 (32.5%)	7 (35%)	20 (33.3%)	0.799
BMI, Kg/m^2^, mean (±SD)	25.1 ± 3.4	24.8 ± 2.8	25 ± 3.2	0.824
ASA score, mean (±SD)	2.5 ± 0.06	2.2 ± 0.04	2.4 ± 0.7	0.856
Pathology				
Malignant	30 (75%)	15 (75%)	45 (75%)	1
PDAC	21	8	29	
IPMN Cancer	3	2	5	
Ampullary Carcinoma	2	2	4	
Cholangiocarcinoma	2	2	4	
Duodenal Carcinoma	1	1	2	
NEC	1	/	1	
Benign	10 (25%)	5 (25%)	15 (25%)	1
IPMN	4	2	6	
Serous cystic Neoplasm	3	1	4	
MCN	2	1	3	
Chronic Pancreatitis	1	1	2	
Tumor size, cm, mean (±SD)	2.86 ± 1.7	2.55 ± 1.4	2.8 ± 1.6	0.822
Neoadjuvant CHT, *n* (%)	6 (15%)	2 (10%)	8 (13.3%)	0.479
Pancreatic texture, *n* (%)				1
Soft	16 (40%)	8 (40%)	24 (40%)	
Hard	24 (60%)	12 (60%)	36 (60%)	
Wirsung duct diameter, median ± SD	3.4 ± 2.4	2.9 ± 2.5	3.2 ± 2.4	0.627
≥3 mm, *n* (%)	31 (77.5%)	14 (70%)	45 (75%)	0.669
<3 mm, *n* (%)	9 (22.5%)	6 (30%)	15 (25%)	0.611
ISGPS classification				
A	19 (47.5%)	9 (45%)	28 (46.7%)	0.821
B	5 (12.5%)	3 (15%)	8 (13.3%)	0.793
C	12 (30%)	5 (25%)	17 (28.3%)	0.645
D	4 (10%)	3 (15%)	7 (11.7%)	0.612
Mean CRS-POPF ± SD	4.6 ± 2.2	5.1 ± 1.8	4.7 ± 2.1	0.433
Histopathology, *n* (%)				
Ampullary/Duodenal/Cystic	8 (20%)	5 (25%)	13 (21.7%)	0.523
PDAC/IPMN/others	32 (80%)	15 (75%)	47 (78.3%)	
Estimated blood loss				
≥500 mL	8 (20%)	4 (20%)	12 (20%)	1
<500 mL	32 (80%)	16 (80%)	48 (80%)	
Categories of POPF risk, *n* (%)				0.788
Negligible	6 (15%)	2 (10%)	8 (13.3%)	
Low	16 (40%)	6 (30%)	22 (%)	
Intermediate	14 (35%)	9 (45%)	23 (35%)	
High	4 (10%)	3 (15%)	7 (11.7%)	

BMI: Body Mass Index, ASA: American Society of Anesthesiologists, PDAC: Pancreatic Ductal Adenocarcinoma, IPMN: Intraductal Papillary Mucinous Neoplasm, NET: Neuroendocrine Cancer, MCN: Mucinous Cystic Neoplasm, CHT: Chemotherapy, CRS: Clinical risk score, POPF: Post-operative pancreatic fistula.

**Table 3:** Postoperative outcomes of patients who underwent PJ vs. PG reconstruction.

Variables	PJ (*n* = 40)	PG (*n* = 20)	Overall (*n* = 60)	*p* Value
Operative time, min, median ± SD	375 ± 102	315 ± 110	355 ± 103	0.345
Time of the anastomoses, min, median ± SD	32 ± 11	25 ± 14	30.2 ± 12	0.002
Estimated blood loss, ml, median (IQR)	270 (180–600)	295 (200–700)	275 (180–600)	0.442
Intraoperative blood transfusion, *n* (%)	3 (7.5%)	1 (5%)	4 (6.7%)	0.766
Post-operative complications, *n* (%)	19 (47.5%)	9 (45%)	28 (46.6%)	0.635
Grade < III	−11 (27.5%)	−6 (30%)	−17 (28.3%)	0.826
Grade ≥ III	−8 (20%)	−3 (15%)	−11 (18.3%)	0.542
Biochemical leak	5 (12.5%)	3 (15%)	8 (13.3%)	0.524
CR-POPF	5 (12.5%)	2 (10%)	7 (11.7%)	0.827
Grade B	−3 (7.5%)	−1 (5%)	−4 (6.7%)	0.789
Grade C	−2 (5%)	−1 (5%)	−3 (5%)	0.977
Delayed gastric emptying, *n* (%)	2 (5%)	1 (5%)	3 (5%)	0.928
Grade C Postoperative hemorrhage, *n* (%)	1 (2.5%)	2 (10%)	3 (5%)	0.338
Pancreatitis, *n* (%)	1 (2.5%)	/	1 (1.4%)	0.782
Bile leakage, *n* (%)	1 (2.5%)	1 (5%)	2 (3.3%)	0.654
Ascites, *n* (%)	1 (2.5%)	/	1 (1.4%)	0.782
Intra-abdominal collection, *n* (%)	3 (7.5%)	/	3 (4.3%)	0.002
Length of hospital stays, days, median ± SD	14 ± 4	11 ± 6	15.8 ± 5	0.223
Readmission, *n* (%)	4 (10%)	1 (5%)	5 (8.3%)	0.524
Reoperation, *n* (%)	2 (5%)	1 (5%)	3 (5%)	0.928
Mortality 90-days, *n* (%)	2 (5%)	1 (5%)	3 (5%)	0.928

CR-POPF: Clinically Relevant Postoperative pancreatic fistula.

**Table 4:** Comparison of postoperative results of PJ and PG cohorts matched for the four variables (histopathology, pancreatic texture, pancreatic duct diameter, intraoperative blood loss) of the clinical risk score for post-operative pancreatic fistula (POPF).

Variables	PJ (*n* = 20)	PG (*n* = 20)	*p* Value
Histopathology, *n* (%)			
- PDAC/IPMN	14 (70%)	15 (75%)	0.855
- Ampullary, Duodenal, Cystic	6 (30%)	5 (25%)	0.793
Pancreatic texture, *n* (%)			
- Soft	13 (65%)	13 (65%)	1
- Hard	7 (35%)	7 (35%)	1
Pancreatic duct diameter, mm, *n* (%)			
- ≥3	13 (65%)	14 (70%)	0.643
- <3	7 (35%)	6 (30%)	0.635
ISGPS Classification			
- A	9 (45%)	9 (45%)	1
- B	3 (15%)	3 (15%)	1
- C	5 (25%)	5 (25%)	1
- D	3 (15%)	3 (15%)	1
Intraoperative blood loss, mL, *n* (%)			
- ≥500	5 (25%)	4 (20%)	0.617
- <500	15 (75%)	16 (80%)	0.539
Median Operative time, min (IQR)	330 (270.2–395.8)	315 (265–382)	0.75
Anastomotic time, min (IQR)	46 (28–52)	25 (18–40)	0.002
Morbidity rate, *n* (%)	11 (55%)	9 (45%)	0.721
- Minor	5 (25%)	6 (30%)	0.586
- Major	6 (30%)	3 (15%)	0.324
Biochemical Leak, *n* (%)	4 (20%)	3 (15%)	0.721
CR–POPF, *n* (%)	3 (15%)	2 (10%)	0.478
Delayed gastric emptying, *n* (%)	1 (5%)	1 (5%)	1
Post-pancreatectomy hemorrhage, *n* (%)	/	2 (10%)	0.003
Intra-abdominal collection, *n* (%)	3 (15%)	/	0.002
Reoperation, *n* (%)	2 (10%)	1 (5%)	0.474
Median length of hospital stays, days (IQR)	14.2 (12.4–22)	11.5 (9.5–19)	0.165

**Table 5:** Risks factors for POPF.

Variables	CR-POPF (*n* = 7)	No-POPF (*n* = 53)	Univariate *p* Value	Odds Ratio	95% CI
Age					
≥65 years	4	27	0.76		
<65 years	3	26			
Sex					
Male	4	36	0.57		
Female	3	17			
BMI					
≥25 Kg/m^2^	5	14	<0.05	6.96	(1.2–40.1)
<25 Kg/m^2^	2	39			
Diabetes					
YES	1	10	0.77		
NO	6	43			
ASA score					
≥3	3	29	0.55		
<3	4	24			
Pancreatic duct diameter					
≥3 mm	2	43			
<3 mm	5	10	<0.05	10.7	(1.8–63.6)
Underlying pathology					
PDAC/IPMN/etc.	4	43	0.16		
Ampullary/Cystic/Duodenal	3	10			
Tumor size					
≥2.5 cm	2	21	0.59		
<2.5 cm	5	33			
Texture of the pancreas					
Soft	6	18	<0.05	11.66	(1.3–104.4)
Hard	1	35		-	
Operative time					
≥355 min	4	34	0.71		
<355 min	3	19			
Blood loss					
≥500 mL	5	7	<0.05	10.95	(2.1–56.3)
<500 mL	2	46		-	
Reconstruction type					
PJ	5	35	0.77		
PG	2	18			

BMI: Body Mass Index, ASA: American Society of Anesthesiologist, PDAC: Pancreatic ductal adenocarcinoma, IPMN: Intraductal Papillary Mucinous Neoplasm, PJ: Pancreatojejunostomy, PG: Pancreatogastrostomy.

## Data Availability

Data are available on request.

## Supplementary Materials

The following are available online at https://www.mdpi.com/article/10.3390/jcm10102181/s1, Video S1: Robotic-assisted PJ vs PG: a video technique; (Part A) pancreato-jejunostomy (PJ) and (Part B) pancreato-gastrostomy (PG).
